# Bibliometric analysis of neuroinflammation and Alzheimer’s disease

**DOI:** 10.3389/fnagi.2024.1423139

**Published:** 2024-07-15

**Authors:** Wenxian Sun, Jin Gong, Shaoqi Li, Pin Wang, Xiaodong Han, Chang Xu, Heya Luan, Ruina Li, Boye Wen, Cuibai Wei

**Affiliations:** ^1^Innovation Center for Neurological Disorders and Department of Neurology, Xuanwu Hospital, Capital Medical University, National Clinical Research Center for Geriatric Diseases, Beijing, China; ^2^College of Integrated Traditional Chinese and Western Medicine, Changchun University of Chinese Medicine, Changchun, China; ^3^School of Biological Science and Medical Engineering, Beihang University, Beijing, China

**Keywords:** neuroinflammation, Alzheimer’s disease, bibliometrics, VOSviewer, CiteSpace, microglia

## Abstract

**Background:**

Alzheimer’s disease (AD) is the predominant cause of dementia on a global scale, significantly impacting the health of the elderly population. The pathogenesis of AD is closely linked to neuroinflammation. The present study employs a bibliometric analysis to examine research pertaining to neuroinflammation and AD within the last decade, with the objective of providing a comprehensive overview of the current research profile, hotspots and trends.

**Methods:**

This research conducted a comprehensive review of publications within the Science Citation Index Expanded of the Web of Science Core Collection Database spanning the years 2014 to 2024. Bibliometric analyses were performed using VOSviewer (version 1.6.19) and CiteSpace (version 6.3.R1) software to visualize data on countries, institutions, authors, journals, keywords, and references.

**Results:**

A total of 3,833 publications on neuroinflammation and AD were included from January 2014 to January 2024. Publications were mainly from the United States and China. Zetterberg, Henrik emerged as the author with the highest publication output, while Edison, Paul was identified as the most cited author. The most productive journal was Journal of Alzheimers Disease, and the most co-cited was Journal of Neuroinflammation. Research hotspot focused on microglia, mouse models, oxidative stress, and amyloid-beta through keyword analysis. Additionally, keywords such as blood–brain barrier and tau protein exhibited prolonged citation bursts from 2022 to 2024.

**Conclusion:**

This study provides a comprehensive review of the last 10 years of research on neuroinflammation and AD, including the number and impact of research findings, research hotspots, and future trends. The quantity of publications in this field is increasing, mainly in the United States and China, and there is a need to further strengthen close cooperation with different countries and institutions worldwide. Presently, research hotspots are primarily concentrated on microglia, with a focus on inhibiting their pro-inflammatory responses and promoting their anti-inflammatory functions as a potential direction for future investigations.

## 1 Introduction

As the global population continues to age, the prevalence of dementia is on the rise. In 2015, an estimated 47 million individuals worldwide were affected by dementia, constituting approximately 5% of the elderly population globally. Projections indicate that this number is expected to increase to 75 million by 2030 and 130 million by 2050 (Prince et al., 2015). This trend will result in significant challenges in terms of disease management and economic burden for individuals, families, and public health services. Alzheimer’s disease (AD) is the predominant etiology of dementia on a global scale, manifesting as a multifaceted neurodegenerative condition typified by the accumulation of amyloid beta plaques and neurofibrillary tangles in the brain (Scheltens et al., 2016).

Growing evidence suggests a correlation between neuroinflammation and the pathophysiology of AD (Heneka et al., 2015; Calsolaro and Edison, 2016). Studies have shown that immune cells in the brain, particularly microglia and astrocytes, are key regulators of the inflammatory response in the central nervous system and are involved in the pathogenesis of AD (Kwon and Koh, 2020). The hyperactivation of these cells, along with the presence of inflammatory molecules, are implicated in the pathogenesis of AD (Uddin et al., 2020). Various immune pathways are crucial in maintaining brain homeostasis and influencing the progression of AD (Ennerfelt and Lukens, 2020). Activation of immune cells can trigger programmed cell death through mechanisms such as cellular pyroptosis, apoptosis, and necroptosis, leading to the release of pro-inflammatory cytokines and chronic neuroinflammation. This inflammatory response is linked to neurodegenerative diseases and exacerbates the symptoms of AD (Rajesh and Kanneganti, 2022). Therefore, it is imperative to elucidate the role of neuroinflammation in the development of AD.

Despite the abundance of literature on neuroinflammation and AD, there is a notable absence of comprehensive overview information regarding the number of relevant publications, countries, authors, institutions, journals, and commonly used keywords in relevant studies. This lack of information makes it challenging to identify research hotspots and emerging research directions in the field. Bibliometrics, as a comprehensive quantitative and qualitative analysis method, can provide valuable insights into various characteristics of publications (Donthu et al., 2021). The utilization of scientific databases, such as Web of Science, has facilitated the advancement of bibliometric research (Hao et al., 2018). In recent years, bibliometrics has become a prevalent method for analyzing advancements in the field of neurology (Wilson et al., 2021). Consequently, employing bibliometric tools, we conducted a comprehensive analysis of literature pertaining to neuroinflammation and AD over the past decade. This analysis included an investigation of publication and citation patterns, prominent authors, institutions, countries, journals, and research hotspots in the fields of neuroinflammation and AD through bibliometric and visualization methodologies. This study provides valuable insight into the overall profile of neuroinflammation and AD research.

## 2 Methods

### 2.1 Data acquisition and search strategy

For this research, the Web of Science Core Collection (WoSCC) database was chosen as the primary data source, with the Science Citation Index Expanded (SCI-E) utilized to ensure thorough and accurate data retrieval. The WoSCC database is widely acknowledged as a prominent online resource for bibliometric analysis, encompassing a diverse array of research disciplines and a substantial quantity of peer-reviewed, reputable journals (Noda et al., 2023; Wang et al., 2023). SCI-E, a sub database within WoSCC dedicated to journal citations, offers a comprehensive multidisciplinary coverage of the natural sciences.

**Figure 1:**
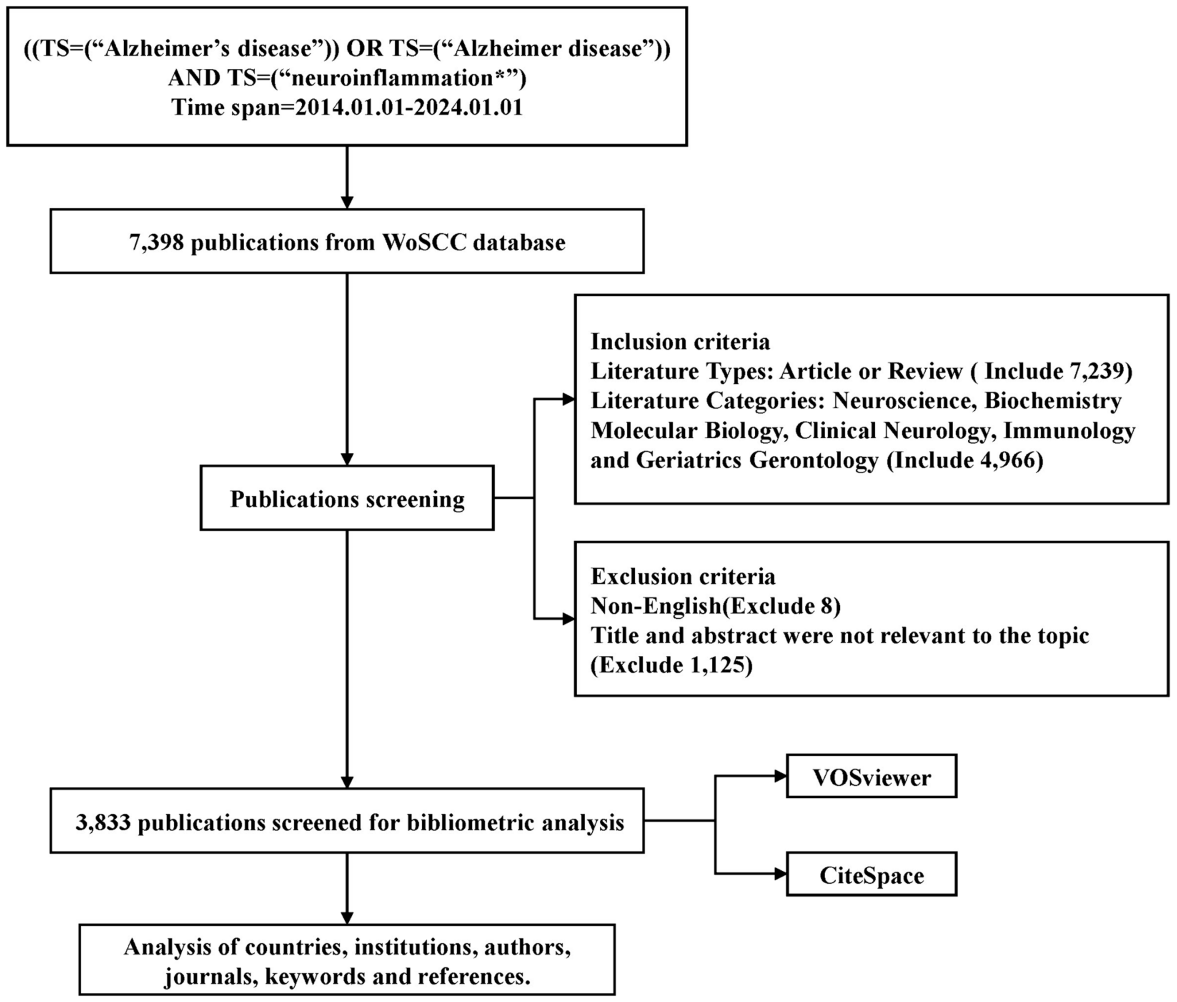
Flowchart for the selection of publications included in this study.

The search terms used to identify publications included topic: (“Alzheimer’s disease” OR “Alzheimer disease”) AND topic: (“neuroinflammation*”). The retrieval period spanned from January 01, 2014 to January 01, 2024. Literature type was limited to articles and review papers. Literature related to the search terms was mainly focused in the fields of Neuroscience, Biochemistry Molecular Biology, Clinical Neurology, Immunology and Geriatrics Gerontology. Subsequently, literature in non-English languages and literature not pertinent to the topic were excluded by reviewing the article title, abstract, and full article. Ultimately, a total of 3,833 papers were selected for inclusion in this study. The detailed search strategy is illustrated in Figure 1. The data from these 3,833 publications were exported as a “plain text file” for further analysis using VOSviewer and CiteSpace.

### 2.2 Data analysis

Bibliometric analyses were conducted utilizing VOSviewer (Version 1.6.19) and CiteSpace (Version 6.3.R1). VOSviewer (van Eck and Waltman, 2010) is a freely available software tool used for visualizing and analyzing authors, keywords, citations, etc., presenting co-occurrence relationships graphically. VOSviewer offers features such as network visualization, overlay visualization, and density visualization. In VOSviewer’s visualization, terms with a closer distance were classified into the same cluster. The size of the nodes within the visualization corresponds to the frequency of occurrence of the terms, while the distance between nodes signifies the strength of their association.

CiteSpace (Synnestvedt et al., 2005) is a scientific literature analysis tool developed by Dr. Chaomei Chen of Drexel University and WISE Lab. The software utilizes co-citation analysis and path-finding network algorithms to visually represent data samples and illustrate the evolution of a particular knowledge domain, enabling the visualization of relationships between literature in the form of scientific knowledge mapping. CiteSpace is capable of identifying research hotspots and trends in popularity across various time periods through keyword burst analysis, analyzing the development trends (Chen and Song, 2019). Thus, CiteSpace facilitates the elucidation of the historical research trajectory, current research status, and hot topics within a specific field, while also providing insights into the prospective direction of field development.

## 3 Results

### 3.1 Publication summary

**Figure 2:**
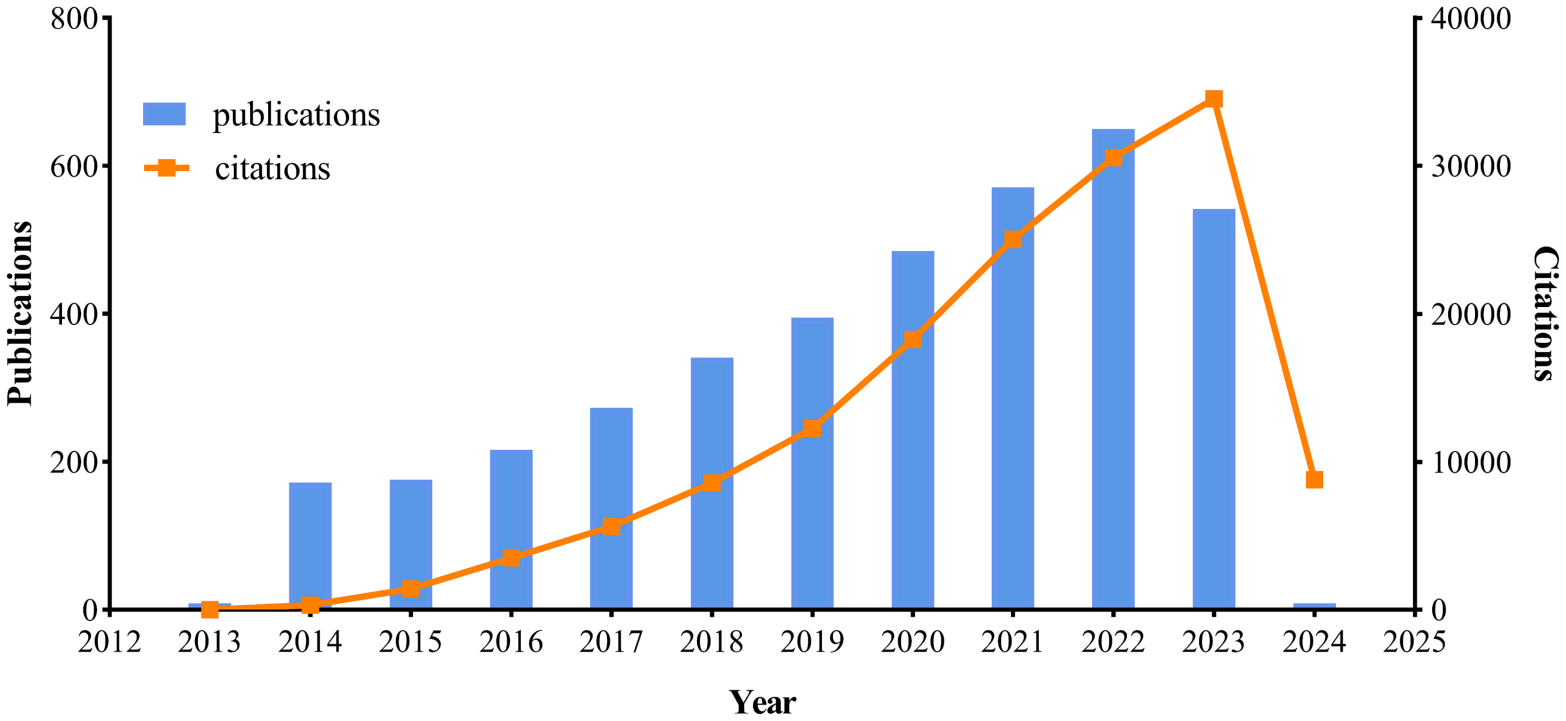
Trends of annual publications and citations.

Over the past decade, a total of 3,833 publications on the topic of neuroinflammation and AD were analyzed, comprising 2,613 articles and 1,220 reviews. Figure 2 illustrated the yearly trends in publications and citations related to neuroinflammation and AD. The number of publications steadily increased each year from 2014 to 2022, reaching a peak of 650 publications in 2022. Although there was a slight decline in publications in 2023 (*n* = 542), the number of citations in that year reached its highest point (*n* = 34,523), suggesting a potential enhancement in the quality of the articles compared to previous years. The most cited publication was “Neuroinflammation in Alzheimer’s disease” by Heneka et al. (2015), with an average of 365.4 citations per year.

### 3.2 Analysis of the countries/regions

**Figure 3:**
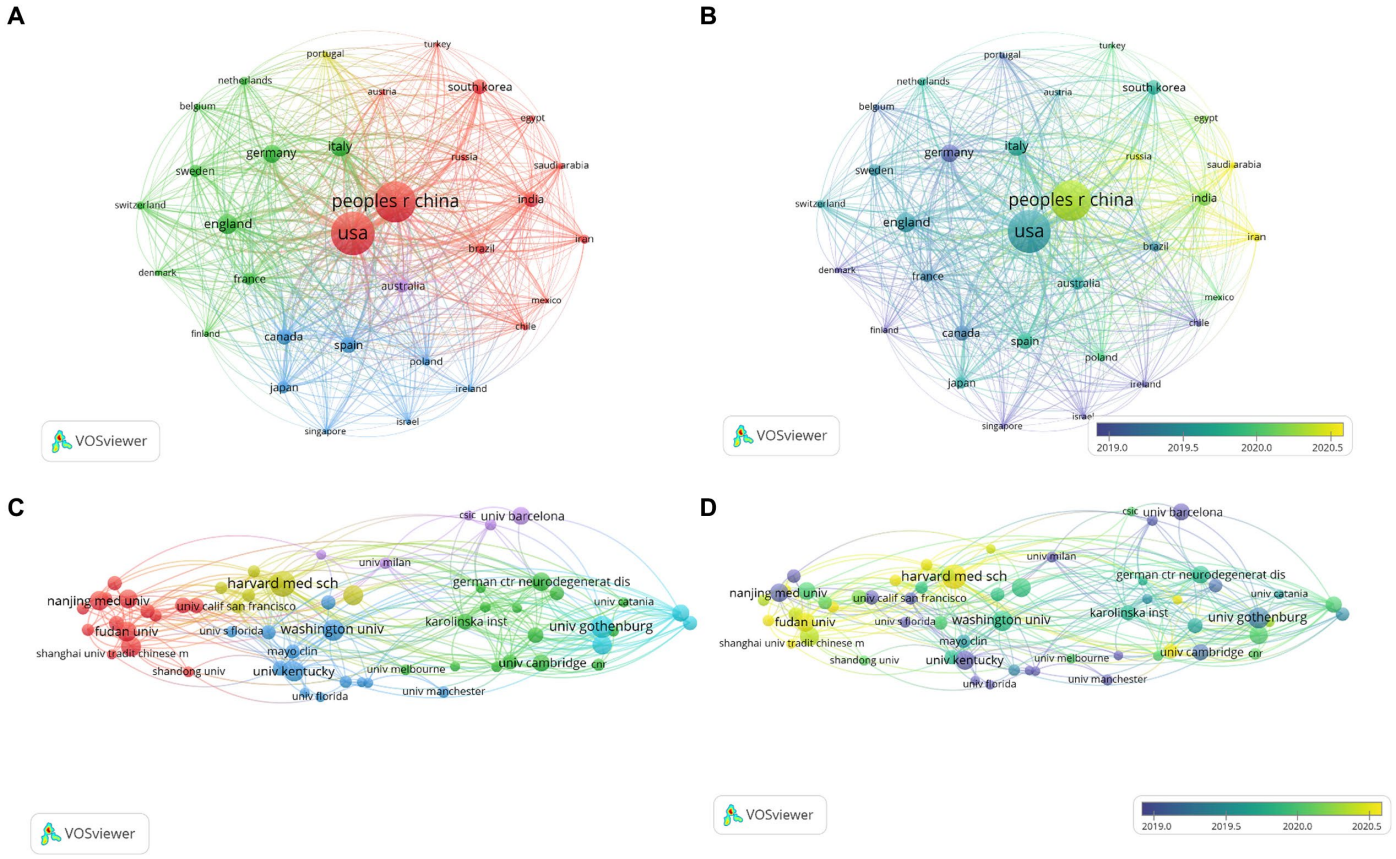
Network visualization map of countries co-authorship analysis **(A)**. Overlay visualization map of countries co-authorship analysis **(B)**. Network visualization map of institutions co-authorship analysis **(C)**. Overlay visualization map of institutions co-authorship analysis **(D)**.

Over the past decade, a total of 3,833 publications on neuroinflammation and AD have been produced across 95 countries/regions. The top 10 countries/regions in terms of publication output and centrality were presented in Tables 1, 2. The majority of publications originated from the USA and China (Figure 3A), with the USA demonstrating the highest influence in terms of publication count (*n* = 1,143), citations (*n* = 56,228), and centrality (0.38). China ranked second in both publication count (*n* = 1,033) and citations (*n* = 32,028), but had a lower centrality score of 0.03. Typically, a centrality value exceeding 0.1 is deemed significant. Only six countries exhibited node centrality values surpassing 0.1, including the USA (0.38), Italy (0.21), England (0.15), Germany (0.14), India (0.13), and Canada (0.12), suggesting that these nations may serve as crucial intermediaries in this domain. In addition, analysis of Figure 3B reveals a delayed initiation of research on neuroinflammation and AD in China compared to the USA.

**Table 1:** Characters of the top 10 countries based on publications.

Rank	Country	Documents	Citations	Centrality
1	USA	1,143	56,228	0.38
2	China	1,033	32,028	0.03
3	England	299	20,035	0.15
4	Italy	282	10,156	0.21
5	Germany	238	16,009	0.14
6	Spain	197	9,257	0.09
7	Canada	191	11,936	0.12
8	India	185	5,714	0.13
9	Korea	170	6,697	0.04
10	Australia	132	6,768	0.06

**Table 2:** Characters of the top 10 countries based on centrality.

Rank	Country	Documents	Citations	Centrality
1	USA	1,143	56,228	0.38
2	Italy	282	10,156	0.21
3	England	299	20,035	0.15
4	Germany	238	16,009	0.14
5	India	185	5,714	0.13
6	Canada	191	11,936	0.12
7	Spain	197	9,257	0.09
8	Sweden	127	8,286	0.08
9	Australia	132	6,768	0.06
10	Saudi Arabia	44	1,198	0.06

### 3.3 Analysis of the institutions

The co-authorship analysis of organizations allows for the estimation of relationships between different institutions based on the number of co-authored publications. A total of 3,856 institutions have published studies on neuroinflammation and AD, with the top 60 institutions included for visual analysis (Figure 3C). Harvard University leaded in both the number of publications (*n* = 59) and citations (*n* = 2,875) in this field, followed by the University of Gothenburg (*n* = 51), the University of Washington (*n* = 49), Fudan University (*n* = 45), and Shanghai Jiao Tong University (*n* = 44). In the overlay network of organizations depicted in the co-authorship analysis (Figure 3D), researchers at the University of Kentucky were pioneers in this research field, while their counterparts at Shanghai University of Traditional Chinese Medicine and Zhejiang University have engaged in more recent investigations in this domain.

### 3.4 Analysis of the authors

**Figure 4:**
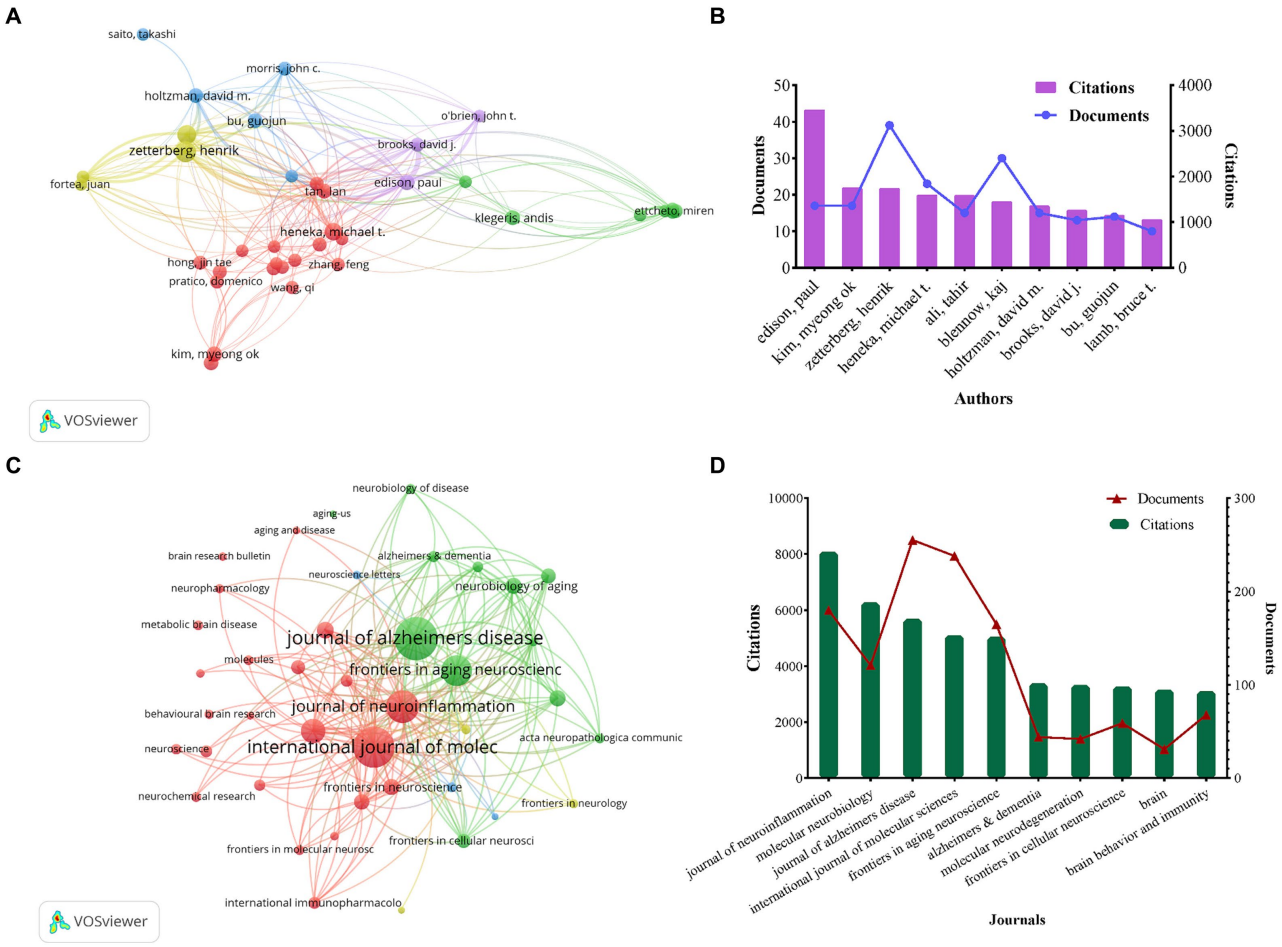
Network visualization map of authors citation analysis **(A)**. The number of citations and publications in the top 10 cited authors **(B)**. Network visualization map of source citation analysis **(C)**. The number of citations and publications in the top 10 cited journals **(D)**.

The full counting citation analysis of authors identified those with high citation counts, as depicted in Figures 4A,B. Ranking authors by the number of citations, Edison, Paul emerged as the most highly cited author with 3,467 citations and 17 documents, followed by Kim, Myeong ok (*n* = 1,755), Zetterberg, Henrik (*n* = 1,750), Heneka, Michael T (*n* = 1,602), and Ali, Tahir (*n* = 1,589). Notably, Zetterberg, Henrik had the highest number of publications among these authors (*n* = 39).

### 3.5 Analysis of the journals

The full counting co-citation analysis of sources revealed the journals with high citation counts, and Figure 4C depicted the journal collaboration mapping created through VOSviewer. The ranking of citations revealed that the Journal of Neuroinflammation was the most cited journal with 8,080 citations and 180 publications, followed by Molecular Neurobiology (*n* = 6,281), Journal of Alzheimers Disease (*n* = 5,690), International Journal of Molecular Sciences (*n* = 5,103) and Frontiers in Aging Neuroscience (*n* = 5,054), respectively. Among them, Journal of Alzheimers Disease had the highest number of publications at 255, as shown in Figure 4D.

### 3.6 Analysis of the keywords

**Figure 5:**
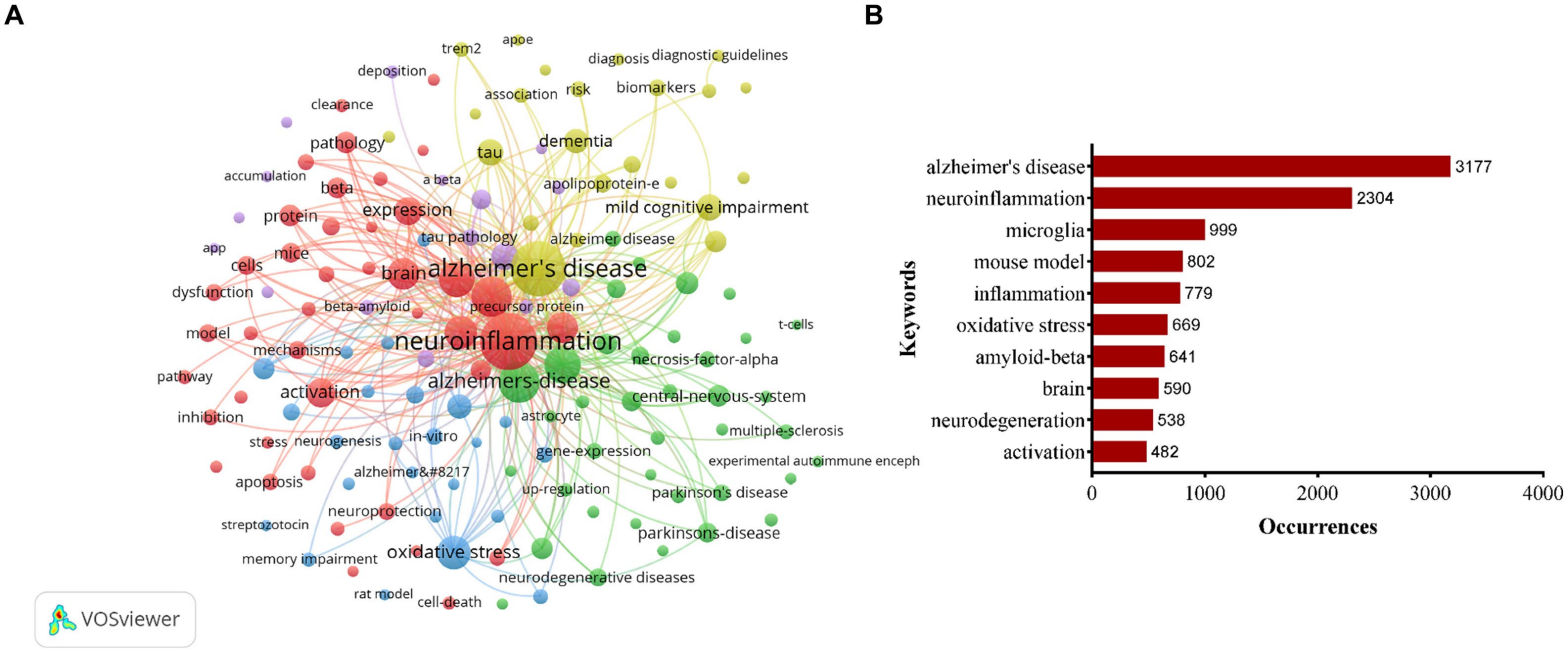
Keywords co-occurrence network visualization map **(A)**. Top 10 keywords with most occurrences in the field of neuroinflammation and Alzheimer’s disease research **(B)**.

Out of 11,736 keywords (with a minimum of 50 occurrences), a total of 143 keywords met the threshold and were included in the analysis. The network visualization in Figure 5A illustrated the relationships among these keywords, with those in closer proximity being grouped into the same cluster. This keywords roughly reflected the primary themes within the field of neuroinflammation and AD research. Specifically, there were five primary clusters identified, with cluster 1 highlighted in red and centered on topics such as neuroinflammation, microglia, and inflammation. Cluster 2, denoted by the color green, was characterized by keywords associated with Alzheimer’s disease, mouse models, and oxidative stress. Cluster 3, depicted in blue, was centered around topics such as oxidative stress, cognitive impairment, memory, and synaptic plasticity. Cluster 4, highlighted in yellow, primarily focused on Alzheimer’s disease, mild cognitive impairment, tau, and dementia. Cluster 5, represented in purple, was dedicated to A-beta, transgenic mice, *in-vivo* studies, amyloid beta, and tau pathology. The most frequently occurring keyword in the clusters was Alzheimer’s disease (*n* = 3,177), followed by neuroinflammation (*n* = 2,304), microglia (*n* = 999), mouse model (*n* = 802), and inflammation (*n* = 779) (Figure 5B).

**Figure 6:**
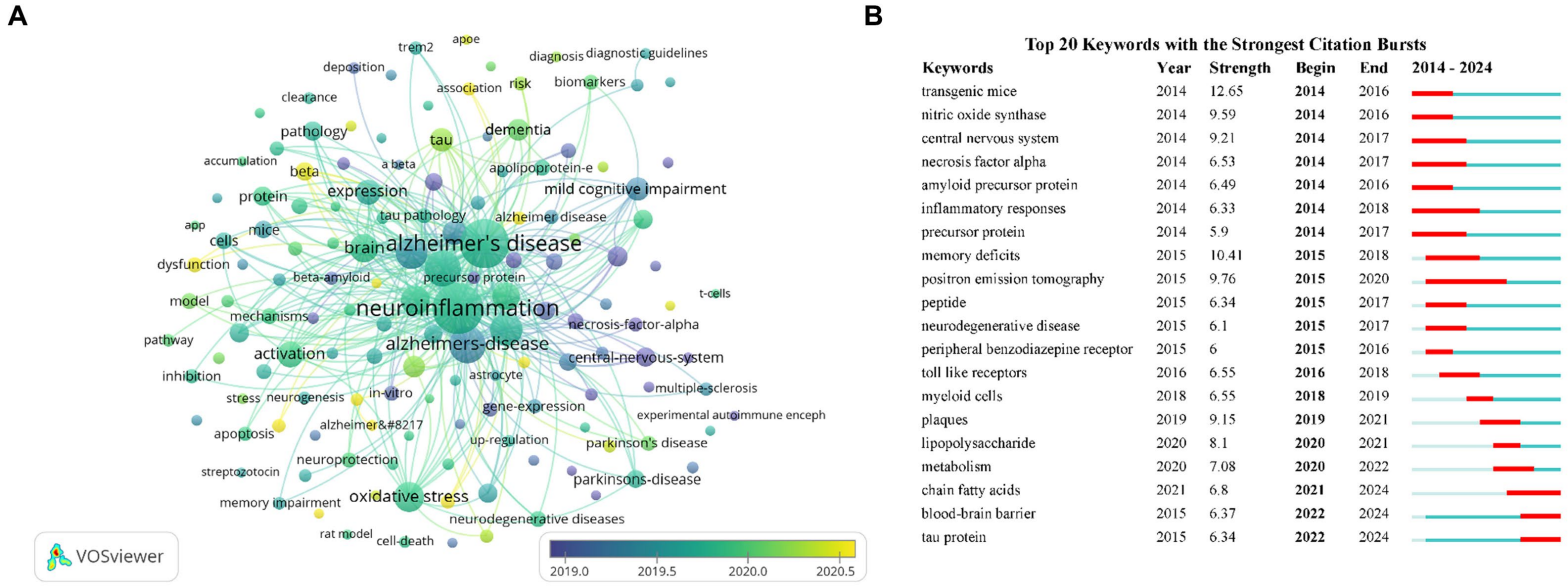
Keywords co-occurrence overlay visualization map **(A)**. The top 20 keywords with the strongest citation bursts based on CiteSpace **(B)**.

Figure 6A presented an overlay visualization of 143 keywords, primarily distributed between the years 2019 and 2020. Keywords appearing earlier were denoted in blue, while those appearing later were denoted in yellow, with yellow markings indicating the most recent research direction. Predominant keywords in the past 2 years have centered on gut microbiota, NLRP3, cognitive decline, autophagy, etc. To analyze the keyword hotspots, we utilized CiteSpace to examine the keyword burst. The top 20 keywords with the strongest citation bursts were identified (Figure 6B). The focus of research hotspots has shifted from topics such as transgenic mice, nitric oxide synthase, central nervous system, and inflammatory responses to positron emission tomography, peptide, toll like receptors, myeloid cells, and plaques, and subsequently to lipopolysaccharide, metabolism, chain fatty acids, blood–brain barrier, and tau protein. Notably, keywords such as blood–brain barrier and tau protein demonstrated sustained bursts from 2022 to 2024.

### 3.7 Analysis of the references

**Figure 7:**
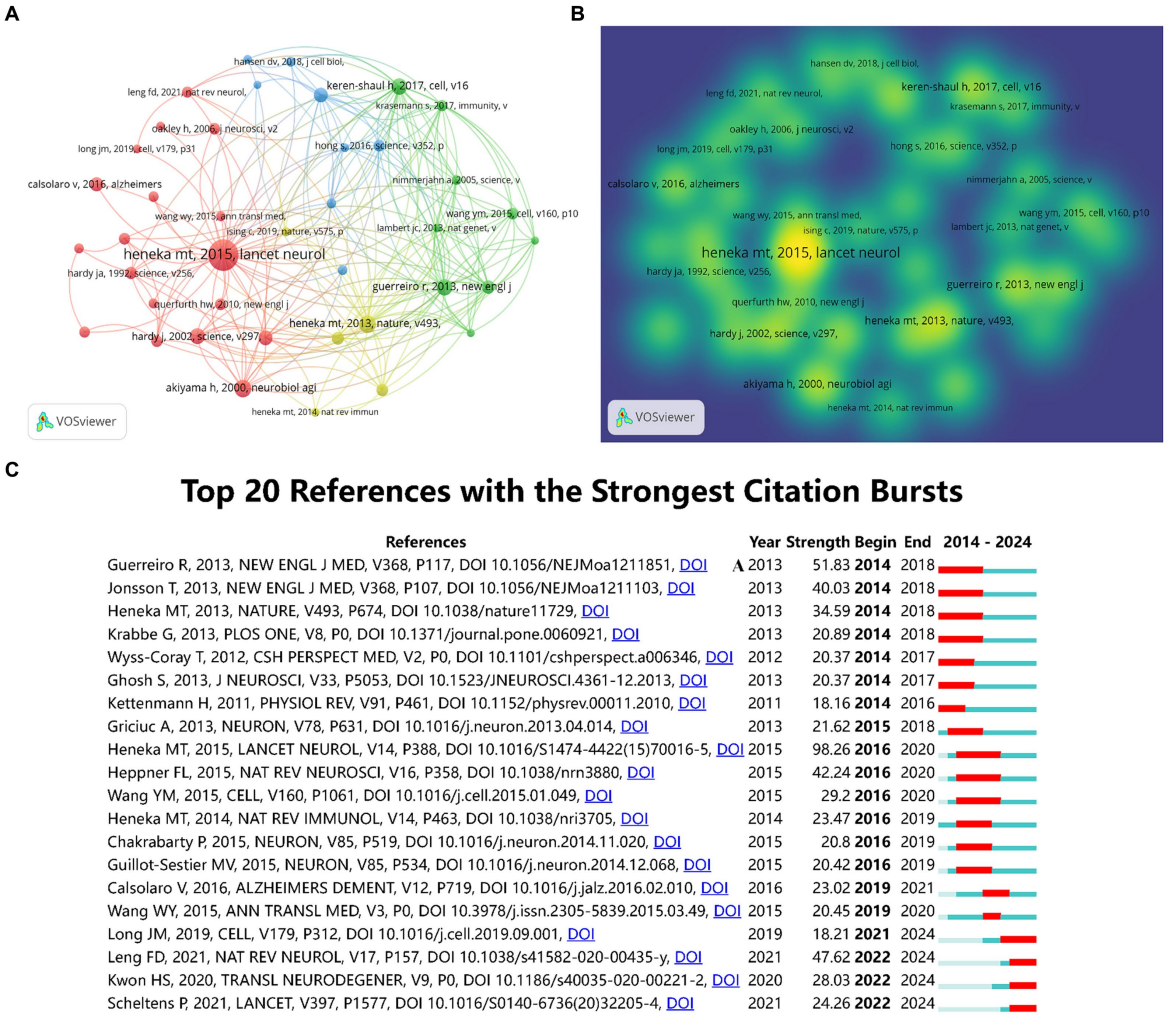
The co-citation network visualization map of references based on VOSviewer **(A)**. The co-citation density visualization map of references **(B)**. The top 20 references with the strongest citation bursts **(C)**.

Over the past decade, a total of 169,797 references have been cited in publications within this field. This analysis focused on the top 40 references, each of which had a minimum of 120 citations. Figures 7A,B presented the network visualization and density visualization of these top 40 co-cited references. The most frequently cited reference was “Neuroinflammation in Alzheimer’s disease (Heneka et al., 2015)” published in Lancet Neurology, followed by “NLRP3 is activated in Alzheimer’s disease and contributes to pathology in APP/PS1 mice (Heneka et al., 2013)” in Nature. both authored by Heneka, Michael T. Figure 7C presented the top 20 references with the strongest citation bursts through CiteSpace. The result identified microglia and astrocytes as hotspots in current research trends.

## 4 Discussion

This study used bibliometric methods to assess the literature on neuroinflammation and AD within the past decade, elucidating the research overview, hotspots and prospective directions within this domain. A comprehensive analysis of 3,833 publications, comprising original articles and reviews released from 2014 to 2024, was conducted. The findings reveal a consistent increase in the volume of published literature over the specified timeframe. Despite a marginal decline in the number of publications in 2023 compared to the preceding years, the citations reached peak during 2023. This suggests that interest in the research field remains strong and anticipates a continued growth in the number of publications in the future.

The quantity of publications serves as a significant metric for assessing the scientific research proficiency of a country, organization, or individual (Garner et al., 2018). The findings of this study indicated that the United States and China held the top two positions in terms of publication volume, underscoring the emphasis placed on research within these two countries. Harvard University in the United States had the highest number of publications, followed by the University of Gothenburg in Sweden. The prolific output of these research institutions is closely linked to state funding and can reflect the level of economic development of the country.

Edison, Paul (Fan et al., 2015, 2017), Zetterberg, Henrik (Panyard et al., 2023; Bjursten et al., 2024), and Heneka, Michael T (Heneka et al., 2010, 2013) have made significant contributions to the advancement of research on neuroinflammation and AD. Edison, Paul et al. hypothesize that there may be two distinct peaks of microglial activation in the trajectory of AD: an initial protective peak followed by a later pro-inflammatory peak. They suggest that anti-microglial agents designed to target the pro-inflammatory phenotype would be most effective in the advanced stages of the disease (Fan et al., 2017). Additionally, their findings indicate that the notable negative correlations observed between cortical levels of microglial activation and decreased glucose metabolism in AD imply that cortical neuroinflammation may be a contributing factor to neuronal impairment in these types of dementias (Fan et al., 2015). Zetterberg, Henrik et al. conducted a comprehensive study on biomarkers in AD, employing extensive proteomic and metabolomic analyses of cerebrospinal fluid. Their findings revealed dysregulation of glucose and carbon metabolism, as well as elevated levels of succinylcarnitine in AD patients (Panyard et al., 2023). Additionally, the researchers identified novel biomarkers indicative of neuroinflammation in AD using a machine learning approach to cerebrospinal fluid proteomics (Gaetani et al., 2021). Heneka, Michael T et al. present compelling evidence of heightened active caspase-1 expression in individuals with mild cognitive impairment and AD, indicating a potential involvement of the inflammasome in the pathogenesis of this neurodegenerative disorder (Heneka et al., 2013). Their findings provide valuable insights for future investigations in this field.

The leading publications in the field included “Journal of Alzheimers Disease” and “International Journal of Molecular Sciences” based on the number of publications, while “Journal of Neuroinflammation” and “Molecular Neurobiology” were the most cited journals. These prominent journals explored the role of astrocytes and microglia in neuroinflammation in AD (Singh, 2022), the impact of gut microbiota dysregulation on the pathogenesis, neuroinflammation and oxidative stress of AD (Liu et al., 2020), and therapeutic approaches targeting neuroinflammation in AD (Yu et al., 2021). The “Journal of Neuroinflammation” concentrates on research pertaining to inflammation in neurological disorders, while the “Journal of Alzheimer’s Disease” focuses on the diagnosis, treatment, biomarker discovery, and other aspects specific to AD within the realm of comprehensive AD specialty journals.

The analysis of keyword frequency serves as a valuable tool for identifying research hotspots and trends. Findings from this investigation indicate that microglia are significantly involved in neuroinflammation and AD research. Microglia are considered to be the resident macrophages of the central nervous system, and microglial phenotypes have been long-established as part of AD pathology (Benitez et al., 2021). A longitudinal study has demonstrated that microglial activation in the initial phases of AD contributes to tissue repair, but as the disease advances, microglia transition to a harmful state, releasing pro-inflammatory molecules that result in neuronal damage (Fan et al., 2017). Disruption of brain homeostasis is observed in individuals with AD, with sustained inflammation driving microglia towards an activated pro-inflammatory M1 phenotype, fostering inflammation and elevating levels of pro-inflammatory cytokines such as TNF-α, IL-4, IL-6, and IL-12 (Martin et al., 2017). Microglia recognize pathogen-associated molecular patterns and damage-associated molecular patterns via various receptors (Toll-like receptors, NOD-like receptors, etc.), activating downstream signaling pathways (NF-κB, MAPK, etc.). This activation leads to the release of inflammatory cytokines and chemokines, which further recruit other immune cells to the site of inflammation (Perry and Holmes, 2014).

The relationship between microglia and Aβ has been extensively demonstrated; however, the specific molecular mechanisms governing their interactions remain elusive. Studies have revealed that excessive production of Aβ by neurons activates the NF-κB pathway in astrocytes, leading to upregulation of extracellular complement C3 expression, ultimately resulting in detrimental effects on neurons and microglia, culminating in neuronal injury and activation of microglia (Lian et al., 2015, 2016). Prolonged stimulation of microglia in proximity to Aβ plaques results in the release of inflammatory mediators during the process of phagocytosis, culminating in excessive microglial activation and exacerbation of the underlying pathology (Michaud et al., 2013). Inflammatory mediators have been shown to upregulate BACE1 activity and NF-κB expression, resulting in the generation of Aβ within the AD brain. Following interaction with microglia and astrocytes, Aβ further stimulates the release of additional inflammatory molecules to the site of inflammation, thereby intensifying neuroinflammation through chemotaxis (Dhapola et al., 2021). This cascade of events contributes to the progression of AD pathology and initiates a detrimental feedback loop.

The extent of research on the relationship between microglia and tau pathology is less comprehensive compared to that of Aβ, and the precise pathogenic mechanism by which microglia promote tau deposition remains uncertain. Microglia can clear abnormal Tau protein through phagocytosis. However, excessively activated microglia may release inflammatory mediators that promote Tau hyperphosphorylation and aggregation, exacerbating neurotoxicity (Laurent et al., 2018). Research indicates that Tau protein can spread between neurons, a process potentially regulated by microglia. Microglia might play a crucial role in the intercellular spread of Tau through the secretion of exosomes and other extracellular vesicles (Asai et al., 2015). Single-cell RNA-seq analysis has shown that tau protein activates the NF-κB signaling pathway to facilitate microglia-mediated tau seeding and diffusion (Wang et al., 2022). In the future, targeting microglia initiation and modulating phenotypic variation may prove to be a promising therapeutic approach for AD.

The analysis of burst detection is a crucial technique for investigating the development of research trends within academic disciplines. Articles or keywords with elevated citation rates suggest that they were prominently discussed or utilized within a defined timeframe. From 2022 onwards, the terms “blood–brain barrier (BBB)” and “tau proteins” have consistently emerged as burst keywords. The etiology of AD is intricately linked to the structural composition of the blood–brain barrier (BBB). The BBB, a meticulously controlled yet delicate tissue framework, is established by endothelial cells in collaboration with pericytes, astrocytes, neurons, and microglia within the neurovascular unit (Liebner et al., 2018). Dysfunction of the BBB initiates a cascade of events involving neuroinflammation and oxidative stress, leading to the accumulation of Aβ. This accumulation further disrupts BBB function, perpetuating a detrimental cycle that ultimately results in cognitive decline (Cai et al., 2018). Within the context of AD, the intricate and interdependent relationship between tau pathology and inflammation is evident. The sustained and heightened inflammatory reactions in glial cells and neurons play a crucial role in exacerbating tau pathology (Chen and Yu, 2023). Investigating the precise mechanism underlying the connection between tau pathology and neuroinflammation holds promise for uncovering novel therapeutic targets for AD.

This study represents the initial attempt to conduct a bibliometric analysis of the past decade of research pertaining to neuroinflammation and AD. However, it is important to acknowledge certain constraints within our study. Firstly, our dataset consists solely of English articles sourced from the WoSCC database, potentially limiting the comprehensiveness of our findings regarding the current landscape of neuroinflammation and AD. Moreover, there is a delay in the accumulation of citations for publications. High-quality publications from recent years may not have the desired number of citations, which may lead to research bias. Finally, the search terms in this study were mainly neuroinflammation and AD, and other related definitions or nouns may not be completely covered.

## 5 Conclusion

This study provides a comprehensive review of the last 10 years of research on neuroinflammation and AD, including the number and impact of research findings, research hotspots, and future trends. The quantity of publications in this field is increasing, mainly in the United States and China, and there is a need to further strengthen close cooperation with different countries and institutions worldwide. Presently, research hotspots are primarily concentrated on microglia, with a focus on inhibiting their pro-inflammatory responses and promoting their anti-inflammatory functions as a potential direction for future investigations.

## Data availability statement

The original contributions presented in the study are included in the article/supplementary material, further inquiries can be directed to the corresponding author.

## Author contributions

WS: Conceptualization, Methodology, Writing – original draft. JG: Methodology, Writing – original draft. SL: Software, Writing – original draft. PW: Formal analysis, Writing – review & editing. XH: Visualization, Writing – original draft. CX: Validation, Writing – review & editing. HL: Investigation, Writing – review & editing. RL: Software, Writing – review & editing. BW: Methodology, Validation, Writing – review & editing. CW: Funding acquisition, Project administration, Supervision, Writing – review & editing.
